# Disentangling Environmental and Within‐Host Drivers of Parasite Dynamics in Natural Populations

**DOI:** 10.1111/mec.70451

**Published:** 2026-07-01

**Authors:** Antoine Perrin, Heinz Richner, Molly Baur, Olivier Glaizot, Philippe Christe

**Affiliations:** ^1^ Department of Ecology and Evolution University of Lausanne Lausanne Switzerland; ^2^ Institute of Ecology and Evolution University of Bern Bern Switzerland; ^3^ Department of Zoology State Museum of Natural Sciences Lausanne Switzerland

**Keywords:** avian malaria, long‐term monitoring, parasite lineage coexistence, *Parus major*, spatio‐temporal dynamics, within‐host competition

## Abstract

Understanding how parasite communities assemble and persist in the wild requires integrating both spatial and temporal dynamics, especially in systems involving multiple co‐circulating lineages. Using a unique 28‐year dataset of individually monitored great tits ( *Parus major* ) from five sites in western Switzerland, we investigated the eco‐evolutionary dynamics of haemosporidian parasites (*Plasmodium* and *Haemoproteus*). We examined (i) spatial and temporal variation in parasite prevalence and lineage composition, (ii) differences in host recovery across lineages, and (iii) within‐host transitions and evidence of competitive interactions. We found significant spatial patterns of prevalence and lineage dominance, not explained by a simple urban–rural gradient, with environmental filtering likely driving local parasite assemblages. While prevalence fluctuated across years, lineage composition remained temporally stable, suggesting long‐term coexistence shaped by site‐specific conditions. Recovery rates, based on whether birds cleared their infection between recaptures, varied across parasite lineages, suggesting lineage‐specific differences in infection persistence. In addition, *Plasmodium homonucleophilum* (SW2 lineage) frequently replaced other *Plasmodium* lineages within hosts but was never itself replaced, consistent with a pattern of competitive exclusion. Our results highlight the importance of spatial heterogeneity, local interactions, and lineage‐specific traits in shaping parasite communities over space and time. This study underscores the value of long‐term, individual‐based monitoring to understand natural parasite dynamics and the processes driving multi‐lineage coexistence.

## 1 Introduction

An important question in parasitology and evolutionary ecology is how the spatial and temporal dynamics of infections shape the long‐term coexistence and turnover of parasite lineages in wild host populations (Soares et al. 2017). In this context, long‐term studies are indispensable for understanding parasite infection dynamics (Yan et al. 2024) and their consequences on host survival and reproduction (Lachish et al. 2011a; Pigeault et al. 2018). They also help identify the abiotic and biotic factors that influence infection outcomes across space and time, such as variation in vector availability, host demography, or climatic conditions (Lachish et al. 2011b; Otero et al. 2019; Samuel et al. 2011). Moreover, long‐term monitoring of parasites in wildlife provides a valuable opportunity to study natural zoonotic host–parasite interactions, whose dynamics in humans are often influenced by control programs and anthropogenic environmental changes.

Among wildlife systems, avian haemosporidian parasites, including *Plasmodium* and *Haemoproteus*, have long served as a model for studying the transmission dynamics of vector‐borne pathogens in natural populations, and they offer important insights for understanding human malaria (Rivero and Gandon 2018). These parasites are globally distributed, infect a wide range of avian hosts, and exhibit diverse lineage‐specific traits such as host specificity, virulence, geographic range and vector associations (LaPointe et al. 2012). Experimental studies have revealed marked differences in virulence among haemosporidian lineages (Palinauskas et al. 2008), and even a single lineage can induce contrasting infection outcomes depending on the host species (Atkinson and LaPointe 2009; Valkiūnas 2005). Haemosporidian community composition shows high overall dissimilarity both within and between biogeographic regions (Ellis et al. 2015; Perrin et al. 2025) and across millennial timescales (Soares et al. 2017), but appears more stable over decadal periods (Bensch et al. 2007). Avian malaria thus provides a relevant system for investigating the spatial and temporal dynamics of multi‐lineage parasite infections in natural host populations.

Within‐host interactions between haemosporidian lineages play a central role in shaping infection dynamics, influencing parasite persistence, competitive exclusion or facilitation, with major consequences for the evolution of virulence (Abkallo et al. 2015; de Roode, Pansini, et al. 2005; Garcia‐Longoria et al. 2022). Experimental studies in rodent malaria models have shown that the competitive outcomes between lineages depend not only on intrinsic factors such as virulence and growth rate (Abkallo et al. 2015; Barclay et al. 2008; Bell et al. 2006; Huijben et al. 2011), but also on extrinsic factors including host genotype (de Roode et al. 2004), immune status (Råberg et al. 2006), and the order of infection, with priority effects often leading to asymmetric outcomes (de Roode, Helinski, et al. 2005; Karvonen et al. 2019). However, most of this evidence comes from laboratory systems that simplify the ecological complexity in which such interactions occur. In natural populations, parasite–parasite interactions are influenced by heterogeneous environmental conditions, fluctuating host immunity, and spatial variation in vector communities and transmission intensity (Clark et al. 2014; Kim and Tsuda 2012). Due to the scarcity of longitudinal data on within‐individual infection dynamics, particularly from recaptured birds in the wild (Knowles et al. 2011), the frequency and consequences of competitive suppression, facilitation, and priority effects under natural conditions remain largely unknown. Yet, such information is crucial for predicting parasite community dynamics and understanding the evolutionary consequences of multi‐lineage infections in wildlife.

In this study, we used a long‐term dataset of individually monitored great tits ( *Parus major* ) sampled over almost three decades (1995–2022) across five sites in western Switzerland. This spatial and temporal coverage allows us to investigate the natural dynamics of haemosporidian infections in the wild, beyond the constraints of laboratory or short‐term field studies. We specifically aimed to (i) quantify spatial and temporal variation in haemosporidian prevalence and community composition, (ii) assess whether host recovery rates differ among the dominant parasite lineages, and (iii) test for non‐random lineage replacement over time within individual hosts. According to Bensch et al. (2007), parasite prevalence is expected to vary over time, possibly following a cyclical pattern, while community composition is expected to remain stable. Pigeault et al. (2024) demonstrated differences in parasitaemia among parasite lineages, which may reflect variation in virulence. Based on this, we hypothesise that such differences could lead to variation in host recovery rates across lineages and that lineages with higher parasitaemia may more frequently replace others within hosts, consistent with competitive dominance by more virulent parasites during intra‐host competition (de Roode, Pansini, et al. 2005).

## 2 Methods

### 2.1 Study Sites and Bird Sampling

This study was conducted on several great tit ( *Parus major* ) populations in western Switzerland. In the canton of Vaud, nest boxes were installed at three locations: Dorigny forest on the campus of the University of Lausanne (46°31′25.607″ N, 6°34′40.714″ E; 380 m altitude), Monod forest in Hautemorges (46°34′19.953″ N, 6°23′59.204″ E; 660 m altitude), and a forest spanning Mont‐la‐Ville and La Praz (46°40′07.27″ N, 6°25′28.35″ E; 920 m altitude). In the canton of Bern, nest boxes were installed in Bremgartenwald (46°57′41.71″ N, 7°24′39.99″ E; 550 m altitude) and Forst forest (46°54′58.70″ N, 7°18′48.83″ E; 630 m altitude) near the town of Bern. The five sites can be arranged along a simple urban–rural gradient: Dorigny and Bremgartenwald are forested areas adjacent to urban environments, whereas Forst, Monod, and La Praz are located in more rural settings.

A total of 3695 birds were captured intermittently across sites and years for blood sampling during the breeding season (April to June) between 1995 and 2022. Bremgartenwald was sampled mainly between 1995 and 2004 (1076 great tits), Forst primarily from 1999 to 2007 (644 samples), La Praz between 2008 and 2022 (259 great tits), and Dorigny and Monod between 2005 and 2022 (1077 and 639 samples, respectively). Blood samples (30–50 μL) were collected from the brachial vein using sterile needles and lithium‐heparin‐lined microvettes. Samples were stored at −20°C in SET buffer until molecular analysis.

### 2.2 Molecular Diagnosis of Haemosporidian Infections

Haemosporidian lineages (*Plasmodium* and *Haemoproteus*) were identified using molecular methods. Total DNA was extracted using the DNeasy Blood & Tissue Kit (Qiagen, Switzerland). A nested PCR protocol was employed to amplify a 478‐bp fragment of the parasite *cytb* gene (Hellgren et al. 2004). Sequencing of PCR products followed van Rooyen et al. (2013), and lineages were identified using the MalAvi database (Bensch et al. 2009). In cases of multiple infections, sequences were resolved visually by inspecting the chromatograms and assigning haplotypes based on the dominant peaks. Ambiguous positions were coded as multiple infections without assigning a specific haplotype.

### 2.3 Data Analyses

#### 2.3.1 Temporal Dynamics of Haemosporidian Prevalence and Community Composition

We assessed variation in haemosporidian prevalence in relation to site and year using a generalised linear mixed model (GLMM) with binomial error, implemented in the *lme4* package in R. Sampling site was included as a fixed factor, and year of capture was treated as a random factor to account for inter‐annual variation. Pairwise site comparisons were derived from model‐estimated marginal means. For site pairs that were never sampled in the same year, contrasts reflect model‐based differences rather than direct within‐year comparisons. Pearson correlation tests evaluated whether the prevalence of the most abundant parasite lineage at each site correlated with total parasite prevalence.

Stability of community composition over time and space was assessed using the Bray–Curtis dissimilarity index. We removed birds that were uninfected or had multiple infections from this analysis. To test the effects of site and year on community composition, we conducted a permutational multivariate analysis of variance (PERMANOVA) using the *adonis2* function from the *vegan* package in R. The model included site and year as explanatory variables, with the Bray–Curtis dissimilarity matrix as the response variable. Marginal testing of each factor was applied using 999 permutations.

#### 2.3.2 Variation in Host Recovery Across Parasite Lineages

We investigated recovery rates of 245 recaptured birds infected by one of the three main lineages in our dataset (*H*. PARUS1 [*H. majoris*], *P*. SGS1 [ *P. relictum* ], and *P*. SW2 [*P. homonucleophilum*]). The spatial and temporal distribution of these recaptured birds across sites and years is presented in Figure S1. Birds were classified as recovered (infection cleared) or not recovered (infection persistent) based on the subsequent capture. A chi‐squared test compared the distribution of recovered and non‐recovered birds among parasite lineages.

#### 2.3.3 Parasite–Parasite Interactions and Lineage Switching

Within‐host parasite dynamics were examined by analysing transitions between *Plasmodium* and *Haemoproteus* infections, as well as lineage switches within the *Plasmodium* genus. We used longitudinal infection histories from 50 birds recaptured at least twice and infected by different haemosporidian lineages. The spatial and temporal distribution of these recaptured birds across sites and years is presented in Figure S2. Binomial tests compared observed transition frequencies against the null hypothesis of random switching (expected probability = 0.5), both overall and in specific pairwise comparisons (e.g., *H*. PARUS1 vs. *P*. SGS1 or *P*. SW2). Although we did not measure virulence directly, we used parasitaemia levels reported by Pigeault et al. (2024) as a proxy. Given the higher parasitaemia of *P*. SW2, we assessed whether this lineage systematically replaced other *Plasmodium* lineages within hosts, consistent with expectations of competitive dominance of more virulent parasites during intra‐host competition.

## 3 Results

### 3.1 Spatial and Temporal Dynamics of Haemosporidian Prevalence

**FIGURE 1:**
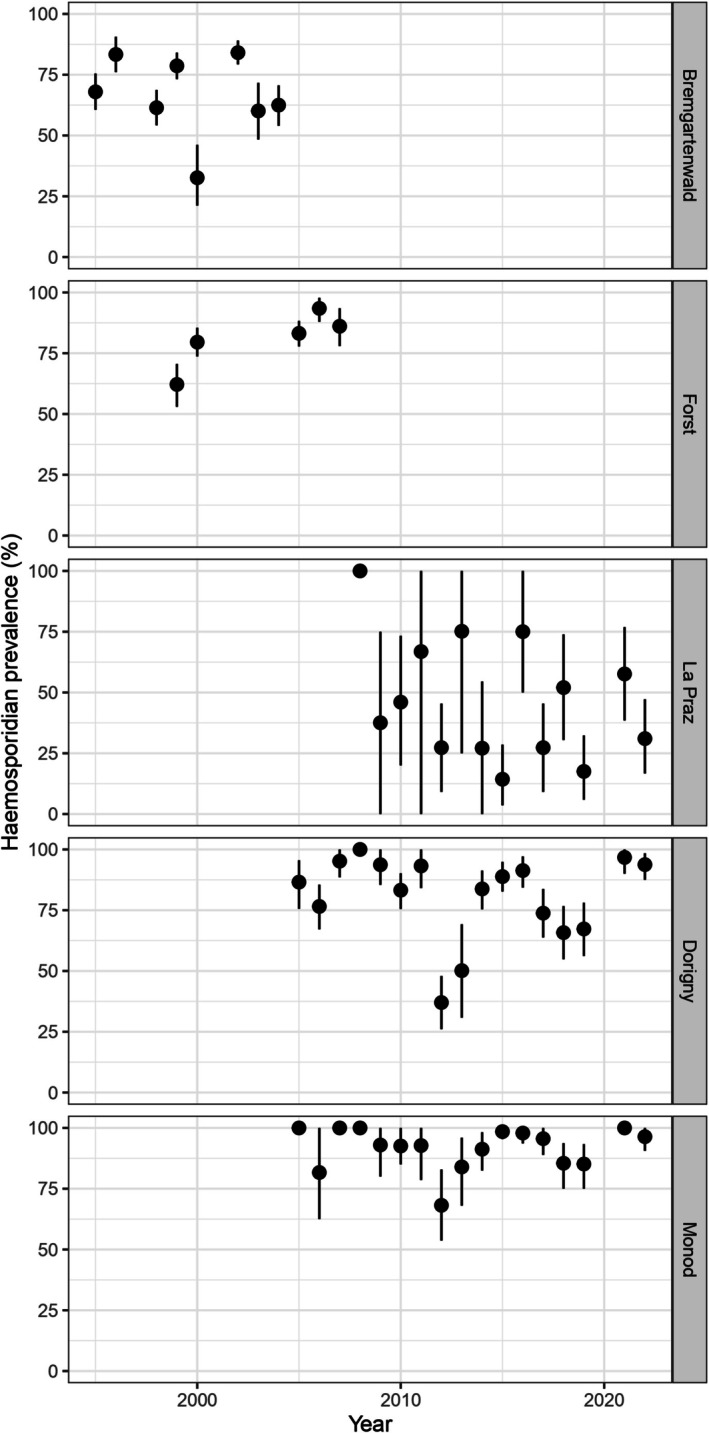
Annual haemosporidian prevalence in five localities in western Switzerland between 1995 and 2022. Points and vertical bars represent means and 95% confidence intervals from the distribution of haemosporidian prevalence in each site for each year and are computed using 1000 bootstraps.

The total haemosporidian prevalence varied significantly among sites (*χ* ^2^ = 284.96, df = 4, *p* < 0.0001; Figure 1). Pairwise comparisons revealed significant differences in prevalence between sites: La Praz had significantly lower prevalence than Dorigny, Forst, and Bremgartenwald, which were all significantly lower than Monod (Table 1). A significant annual variation in haemosporidian prevalence was also observed (*χ* ^2^ = 181.96, df = 1, *p* < 0.0001; Figure 1). Due to the low prevalence observed at La Praz, this site was excluded from the analysis of haemosporidian community composition.

**TABLE 1:** Pairwise comparisons of sites (Bremgartenwald, Dorigny, Forst, La Praz and Monod) based on Tukey's post hoc test. The table presents the estimated differences of haemosporidian prevalence between sites (Estimate), their standard errors (SE), and *p*‐values. Significant pairwise differences (*p* < 0.05) are highlighted in bold.

Comparison	Estimate	SE	*p*
Bremgartenwald—Dorigny	−0.3433	0.265	0.6933
Bremgartenwald—Forst	−0.3806	0.190	0.2666
**Bremgartenwald—La Praz**	**1.6944**	**0.306**	**< 0.0001**
**Bremgartenwald—Monod**	**−1.4936**	**0.297**	**< 0.0001**
Dorigny—Forst	−0.0374	0.223	0.9998
**Dorigny—La Praz**	**2.0376**	**0.172**	**< 0.0001**
**Dorigny—Monod**	**−1.1503**	**0.170**	**< 0.0001**
**Forst—La Praz**	**2.0750**	**0.273**	**< 0.0001**
**Forst—Monod**	**−1.1130**	**0.262**	**0.0002**
**La Praz—Monod**	**−3.1880**	**0.214**	**< 0.0001**

**FIGURE 2:**
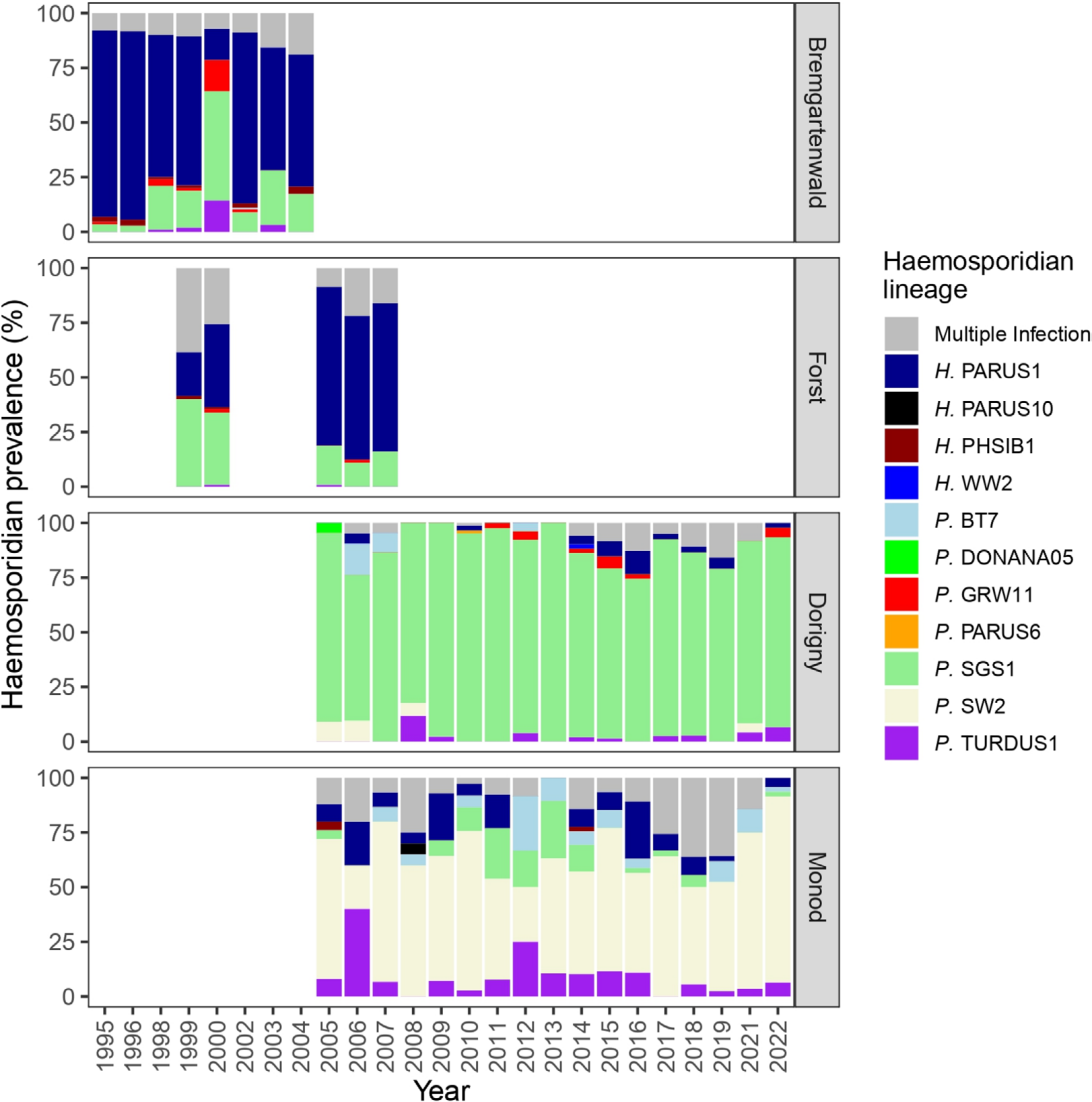
Haemosporidian community composition in five localities in western Switzerland between 1995 and 2022. *H*, *Haemoproteus*; *P*, *Plasmodium*.

Regarding the dominant haemosporidian lineages at each site, *H*. PARUS1 was most prevalent at Bremgartenwald and Forst, whereas *P*. SGS1 dominated at Dorigny and *P*. SW2 was predominant at Monod (Figure 2). Total prevalence was significantly correlated with the prevalence of the dominant parasite lineage within each site. At Dorigny, total prevalence was strongly associated with *P*. SGS1 (*r* = 0.91, *p* < 0.0001), while at Monod, it was associated with *P*. SW2 (*r* = 0.80, *p* = 0.0001). Similarly, total prevalence was linked to *H*. PARUS1 at Bremgartenwald (*r* = 0.93, *p* = 0.002) and Forst (*r* = 0.78, *p* = 0.02).

### 3.2 Variation in Haemosporidian Community Composition

The variation in parasite community composition was primarily explained by sampling site (*F* = 22.00, *R* ^2^ = 0.50, *p* = 0.001). In contrast, the effect of sampling year on haemosporidian community composition was not significant (*F* = 1.06, *R* ^2^ = 0.19, *p* = 0.37). These results highlight the dominant role of spatial variation in shaping parasite communities, while temporal variation within sites was comparatively minor (Figure 2).

### 3.3 Variation in Host Recovery Across Parasite Lineages

Recovery rates differed significantly among the birds infected by the three haemosporidian lineages (*χ* ^2^ = 6.41, df = 2, *p* = 0.04, Table 2), with the primary driver being differences between *P*. SGS1 and *P*. SW2 infections. Birds infected with *P*. SGS1 were less likely to remain infected upon recapture (71%), while in birds infected with *P*. SW2, the parasite exhibited higher persistence rates (90%). For *H*. PARUS1, we found an intermediate persistence rate (82%).

**TABLE 2:** Number of birds that either recovered or remained infected upon recapture for the three main haemosporidian lineages.

Lineage	Recovered	Infected
*Haemoproteus* PARUS1	17	80
*Plasmodium* SGS1	34	85
*Plasmodium* SW2	3	26

### 3.4 Parasite–Parasite Interactions and Lineage Switching

No significant pattern of transitions was observed between *Plasmodium* and *Haemoproteus* infections, either for total prevalence (15 vs. 17, *p* = 0.86) or specific pairwise comparisons (*H*. PARUS1 vs. *P*. SGS1: 8 vs. 10, *p* = 0.81; *H*. PARUS1 vs. *P*. SW2: 4 vs. 5, *p* = 1). Within the *Plasmodium* genus, however, *P*. SW2 systematically replaced other lineages (*P*. BT7, *P*. SGS1 or *P*. TURDUS1) but was not replaced itself (16 vs. 0, *p* < 0.0001).

## 4 Discussion

Our study revealed strong spatial variation in haemosporidian prevalence and parasite community composition across five great tit populations in western Switzerland. While parasite prevalence is often reported to be higher in urban environments compared to more natural habitats (Brearley et al. 2013; Gottdenker et al. 2014; White 2015), there are numerous exceptions, even within the same host–parasite systems such as those involving avian haemosporidian parasites (e.g., Bailly et al. 2016; Evans et al. 2009; Geue and Partecke 2008; Santiago‐Alarcon et al. 2019, 2020). In our study, haemosporidian prevalence did not follow a simple urban–rural gradient. One possible explanation for this pattern is the influence of local ecological conditions linked to elevation. In particular, variation in vector abundance can be strongly influenced by altitude and is vector species specific (Asigau et al. 2017; Dhimal et al. 2014; Eisen et al. 2008). In addition, landscape composition and the relative proportion of structural elements such as forest or agricultural land are known to modulate vector presence and density, with some species being favoured and others negatively affected by certain habitat types (Perrin et al. 2022; Perrin, Schaffner, et al. 2023). This environmental filtering through vector communities may contribute to the spatial distribution of haemosporidian prevalence and lineage composition in our study sites. More broadly, meteorological conditions such as temperature and precipitation can influence haemosporidian transmission through multiple pathways, including effects on vector abundance (Khasnis and Nettleman 2005; Roiz et al. 2014; Talla et al. 2014), survival and feeding activity (Garamszegi 2011; Rose et al. 2020), as well as on parasite development within the vector (Liu‐Helmersson et al. 2014; Paaijmans et al. 2009; Suh et al. 2020), ultimately shaping local prevalence patterns (Cornuault et al. 2013; Loiseau et al. 2013; Pérez‐Rodríguez et al. 2013; Sehgal et al. 2011).

Spatial variation in haemosporidian prevalence may also be related to differences in lineage dominance between sites. Previous studies have shown that haemosporidian lineages differ in their prevalence in relation to landscape structure (Loiseau et al. 2010; Perrin, Khimoun, et al. 2023), supporting the idea that haemosporidian prevalence reflects a lineage‐specific rather than a uniform response to landscape features. This is consistent with recent findings showing that the spatial responses of parasite communities are context dependent (Fecchio et al. 2021), and that changes in parasite prevalence are inherently difficult to predict due to the complexity of host‐vector‐parasite‐environment interactions (Tamayo‐Quintero et al. 2025).

We also detected significant year‐to‐year variation in haemosporidian prevalence. This finding is consistent with other long‐term studies showing that infection rates can fluctuate annually in response to factors such as environmental conditions (e.g., summer temperature), host assemblage, or changes in vector abundance (Bensch et al. 2007; Samuel et al. 2011; Soares et al. 2017; Wilkinson et al. 2016). However, the community composition of haemosporidian lineages within sites appeared relatively stable over time, suggesting that lineage turnover tends to occur at broader evolutionary or biogeographic timescales (Bensch et al. 2007; Soares et al. 2017). This temporal stability of lineage assemblages over decades, despite short‐term fluctuations in prevalence, also suggests a dynamic equilibrium within each local parasite community. Environmental fluctuations may modulate the relative transmission success of lineages from year to year, but long‐term coexistence appears to be maintained at the landscape scale by local ecological conditions.

Beyond the study of the spatial and temporal stability of the prevalence and composition of haemosporidian parasite community, our longitudinal data allowed us to examine variation in host recovery across parasite lineages. Birds infected with *P*. SGS1 were more likely to become PCR‐negative between capture events than those infected with *P*. SW2, which showed higher rates of persistence. The absence of molecular detection does not necessarily indicate complete parasite clearance, as infections may persist at levels below the detection threshold and potentially relapse under certain conditions. Nevertheless, the contrasting patterns of detectability over time suggest lineage‐specific differences in infection dynamics, potentially reflecting variation in virulence or immune evasion strategies. A field study conducted during 3 years in two of our study populations (Dorigny and Monod) has shown that *P*. SW2 induced higher parasite loads than *P*. SGS1 (Pigeault et al. 2024), which could contribute to its increased persistence. Alternatively, *P*. SGS1 may trigger stronger or more effective host immune responses leading to faster clearance. Differences in infection persistence are ecologically and evolutionarily relevant, as chronic infections can enhance transmission opportunities but may also impose greater costs on the host. Persistent lineages may also have a competitive advantage in environments with low vector availability or short transmission seasons.

In this context, we found no significant evidence of directional transitions between *Haemoproteus* and *Plasmodium* lineages within individuals, suggesting limited competition or interaction between these genera in this system. The literature shows no consistent pattern either. One of the few examples even reports a positive association between *Haemoproteus* and *Plasmodium* infections, with parasitaemia of the two genera positively correlated (Garcia‐Longoria et al. 2022). This contrasts with the strong within‐host competition often observed among *Plasmodium* lineages, where total parasite load does not necessarily increase with multiple infections (Bushman et al. 2016; de Roode et al. 2003). In our study, the lineage *P*. SW2 can replace others (e.g., *P*. SGS1, *P*. BT7) but was never itself replaced. This pattern is consistent with a scenario of competitive exclusion, in which *P*. SW2 may outcompete other lineages during co‐infection or more efficiently establish chronic infections that prevent subsequent colonisation. Such competitive dominance could be linked to differences in host specificity. Both *P*. SGS1 and *P*. BT7 are considered generalist parasites with broad host ranges (de la Puente et al. 2021; Valkiūnas 2005), which may involve trade‐offs in within‐host performance. In contrast, if *P*. SW2 exhibits greater host specificity or local adaptation, this could enhance its competitive ability and persistence within great tits.

Competitive exclusion within hosts is well documented in experimental malaria models, where mixed infections often occur less frequently than expected by chance, suggesting that one lineage excludes or suppresses the other (Richie 1988). Several factors influence these outcomes. For instance, slow‐growing lineages can outcompete fast‐growing ones through immune‐mediated mechanisms (Abkallo et al. 2015), while more virulent clones often dominate avirulent ones (Barclay et al. 2008; Bell et al. 2006; de Roode, Pansini, et al. 2005; Råberg et al. 2006; de Roode et al. 2004). Host and competitor genotype also modulate the magnitude of competitive suppression (Huijben et al. 2011; de Roode et al. 2004), and prior residency is a critical determinant of competitive outcome (de Roode, Helinski, et al. 2005; Karvonen et al. 2019). Our results suggest that similar processes may operate in natural systems, and the apparent inability of any lineage to replace *P*. SW2 may reflect its combined advantage in competitive dominance during intra‐host competition and long‐term persistence within hosts.

Despite competitive hierarchies at the individual level, the coexistence of multiple parasite lineages across different locations and over time highlights the importance of spatial heterogeneity (e.g., differences in environment, vector communities, and host populations between sites) and temporal heterogeneity (e.g., year‐to‐year variation in environmental conditions and host‐vector dynamics) in maintaining parasite diversity. For instance, *P*. SW2 was almost exclusively detected in Monod, which likely reflects differences in vector community composition. Glaizot et al. (2012) reported significant differences in mosquito species assemblages between Dorigny and Monod. While *Culex pipiens* , the known vector of *P*. SGS1 (Lalubin et al. 2013), is common at both sites, and *P*. SW2 has occasionally been detected in this species (Glaizot et al. 2012), it does not complete its development in *C. pipiens* (Valkiūnas 2005). Instead, it may rely on alternative vectors such as *Culiseta* spp., which are more abundant in Monod (Glaizot et al. 2012). Such vector constraints may restrict *P*. SW2 transmission in specific areas. Additionally, immigration of other lineages via migratory hosts or vectors, along with negative‐frequency dependent selection, likely contribute to maintaining parasite diversity despite the competitive advantage of *P*. SW2. Our results support a model where local competition and infection persistence shape within‐host dynamics, while environmental and temporal variation allow multiple lineages to coexist across the landscape. Future studies integrating vector monitoring and host immune responses will be critical to understanding the drivers of lineage persistence and parasite competition over space and time. In addition, laboratory infections can help determine host competence for specific parasite lineages and clarify intra‐host dynamics under controlled conditions.

In conclusion, this study illustrates the value of long‐term, individual‐based monitoring for understanding the spatial and temporal dynamics of parasite communities in natural populations. It also enables detailed investigations into host–parasite interactions, including lineage‐specific traits, recovery dynamics, and within‐host processes. Such datasets are essential for understanding the mechanisms driving parasite persistence and coexistence, and more broadly, the ecological and evolutionary dynamics of parasites in the wild.

## Author Contributions

A.P., H.R., O.G. and P.C. contributed to the study conception and design. Material preparation and data collection were managed by H.R., O.G. and P.C. A.P. performed data analysis, data interpretation and wrote the first draft of the manuscript. M.B. conducted the laboratory work. All authors improved the drafts and approved the final manuscript.

## Funding

This work was supported by the Swiss National Science Foundation (grants 31003A_120479, 31003A_138187, 31003A_159600, 31003A_179378 to Philippe Christe, and 31003A_34020, 31003A_43570, 31003A_53956, 31003A_102017, 31003A_122566 to Heinz Richner).

## Conflicts of Interest

The authors declare no conflicts of interest.

## Supporting information


**Figure S1:** Distribution of bird captures across sampling sites and years for the 245 recaptured birds infected by one of the three main lineages in our dataset (H. PARUS1 [*H. majoris*], P. SGS1 [ *P. relictum* ], and P. SW2 [*P. homonucleophilum*]).
**Figure S2:** Distribution of bird captures across sampling sites and years for the 50 birds recaptured at least twice and infected by different haemosporidian lineages.

## Data Availability

Raw data, including all identified lineages and associated metadata, are available in the Mendeley repository (DOI: https://doi.org/10.17632/sn6r54y4c6.1) and on the MalAvi database.

## References

[mec70451-bib-0001] Abkallo, H. M. , J.‐A. Tangena , J. Tang , et al. 2015. “Within‐Host Competition Does Not Select for Virulence in Malaria Parasites: Studies With *Plasmodium yoelii* .” PLoS Pathogens 11, no. 2: e1004628. 10.1371/journal.ppat.1004628. 25658331 PMC4450063

[mec70451-bib-0002] Asigau, S. , D. A. Hartman , J. M. Higashiguchi , and P. G. Parker . 2017. “The Distribution of Mosquitoes Across an Altitudinal Gradient in the Galapagos Islands.” Journal of Vector Ecology 42, no. 2: 243–253. 10.1111/jvec.12264. 29125252

[mec70451-bib-0003] Atkinson, C. T. , and D. A. LaPointe . 2009. “Introduced Avian Diseases, Climate Change, and the Future of Hawaiian Honeycreepers.” Journal of Avian Medicine and Surgery 23, no. 1: 53–63. 19530408 10.1647/2008-059.1

[mec70451-bib-0004] Bailly, J. , R. Scheifler , M. Belvalette , et al. 2016. “Negative Impact of Urban Habitat on Immunity in the Great Tit *Parus major* .” Oecologia 182, no. 4: 1053–1062. 10.1007/s00442-016-3730-2. 27646717

[mec70451-bib-0005] Barclay, V. C. , L. Råberg , B. H. K. Chan , S. Brown , D. Gray , and A. F. Read . 2008. “CD4+T Cells Do Not Mediate Within‐Host Competition Between Genetically Diverse Malaria Parasites.” Proceedings of the Royal Society B: Biological Sciences 275, no. 1639: 1171–1179. 10.1098/rspb.2007.1713. PMC2373868 18292054

[mec70451-bib-0006] Bell, A. S. , J. C. D. Roode , D. Sim , and A. F. Read . 2006. “Within‐Host Competition in Genetically Diverse Malaria Infections: Parasite Virulence and Competitive Success.” Evolution 60, no. 7: 1358–1371. 10.1111/j.0014-3820.2006.tb01215.x. 16929653

[mec70451-bib-0007] Bensch, S. , O. Hellgren , and J. Pérez‐Tris . 2009. “MalAvi: A Public Database of Malaria Parasites and Related Haemosporidians in Avian Hosts Based on Mitochondrial Cytochrome b Lineages.” Molecular Ecology Resources 9, no. 5: 5. 10.1111/j.1755-0998.2009.02692.x. 21564906

[mec70451-bib-0008] Bensch, S. , J. Waldenström , N. Jonzán , et al. 2007. “Temporal Dynamics and Diversity of Avian Malaria Parasites in a Single Host Species.” Journal of Animal Ecology 76, no. 1: 112–122. 17184359 10.1111/j.1365-2656.2006.01176.x

[mec70451-bib-0009] Brearley, G. , J. Rhodes , A. Bradley , et al. 2013. “Wildlife Disease Prevalence in Human‐Modified Landscapes.” Biological Reviews 88, no. 2: 427–442. 10.1111/brv.12009. 23279314

[mec70451-bib-0010] Bushman, M. , L. Morton , N. Duah , et al. 2016. “Within‐Host Competition and Drug Resistance in the Human Malaria Parasite Plasmodium Falciparum.” Proceedings of the Royal Society B: Biological Sciences 283, no. 1826: 20153038. 10.1098/rspb.2015.3038. PMC4810865 26984625

[mec70451-bib-0011] Clark, N. J. , S. M. Clegg , and M. R. Lima . 2014. “A Review of Global Diversity in Avian Haemosporidians (*Plasmodium* and *Haemoproteus*: Haemosporida): New Insights From Molecular Data.” International Journal for Parasitology 44, no. 5: 5. 10.1016/j.ijpara.2014.01.004. 24556563

[mec70451-bib-0012] Cornuault, J. , A. Khimoun , R. J. Harrigan , et al. 2013. “The Role of Ecology in the Geographical Separation of Blood Parasites Infecting an Insular Bird.” Journal of Biogeography 40, no. 7: 1313–1323. 10.1111/jbi.12098.

[mec70451-bib-0013] de la Puente, J. M. , D. Santiago‐Alarcon , V. Palinauskas , and S. Bensch . 2021. “Plasmodium Relictum.” Trends in Parasitology 37, no. 4: 355–356. 10.1016/j.pt.2020.06.004. 32660871

[mec70451-bib-0014] de Roode, J. C. , R. Culleton , S. J. Cheesman , R. Carter , and A. F. Read . 2004. “Host Heterogeneity Is a Determinant of Competitive Exclusion or Coexistence in Genetically Diverse Malaria Infections.” Proceedings of the Royal Society B: Biological Sciences 271, no. 1543: 1073. 10.1098/rspb.2004.2695. PMC1691691 15293862

[mec70451-bib-0015] de Roode, J. C. , M. E. H. Helinski , M. A. Anwar , and A. F. Read . 2005. “Dynamics of Multiple Infection and Within‐Host Competition in Genetically Diverse Malaria Infections.” American Naturalist 166, no. 5: 531–542. 10.1086/491659. 16224719

[mec70451-bib-0016] de Roode, J. C. , R. Pansini , S. J. Cheesman , et al. 2005. “Virulence and Competitive Ability in Genetically Diverse Malaria Infections.” Proceedings of the National Academy of Sciences 102, no. 21: 7624–7628. 10.1073/pnas.0500078102. PMC1140419 15894623

[mec70451-bib-0017] de Roode, J. C. , A. F. Read , B. H. K. Chan , and M. J. Mackinnon . 2003. “Rodent Malaria Parasites Suffer From the Presence of Conspecific Clones in Three‐Clone *Plasmodium chabaudi* Infections.” Parasitology 127, no. 5: 411–418. 10.1017/S0031182003004001. 14653530

[mec70451-bib-0018] Dhimal, M. , B. Ahrens , and U. Kuch . 2014. “Species Composition, Seasonal Occurrence, Habitat Preference and Altitudinal Distribution of Malaria and Other Disease Vectors in Eastern Nepal.” Parasites & Vectors 7, no. 1: 540. 10.1186/s13071-014-0540-4. 25430654 PMC4252987

[mec70451-bib-0019] Eisen, L. , B. G. Bolling , C. D. Blair , B. J. Beaty , and C. G. Moore . 2008. “Mosquito Species Richness, Composition, and Abundance Along Habitat‐Climate‐Elevation Gradients in the Northern Colorado Front Range.” Journal of Medical Entomology 45, no. 4: 800–811. 10.1093/jmedent/45.4.800. 18714885

[mec70451-bib-0020] Ellis, V. A. , M. D. Collins , M. C. I. Medeiros , et al. 2015. “Local Host Specialization, Host‐Switching, and Dispersal Shape the Regional Distributions of Avian Haemosporidian Parasites.” Proceedings of the National Academy of Sciences 112, no. 36: 11294–11299. 10.1073/pnas.1515309112. PMC4568705 26305975

[mec70451-bib-0021] Evans, K. L. , K. J. Gaston , S. P. Sharp , A. McGowan , M. Simeoni , and B. J. Hatchwell . 2009. “Effects of Urbanisation on Disease Prevalence and Age Structure in Blackbird *Turdus merula* Populations.” Oikos 118, no. 5: 774–782. 10.1111/j.1600-0706.2008.17226.x.

[mec70451-bib-0022] Fecchio, A. , N. J. Clark , J. A. Bell , et al. 2021. “Global Drivers of Avian Haemosporidian Infections Vary Across Zoogeographical Regions.” Global Ecology and Biogeography 30, no. 12: 2393–2406. 10.1111/geb.13390.

[mec70451-bib-0023] Garamszegi, L. Z. 2011. “Climate Change Increases the Risk of Malaria in Birds.” Global Change Biology 17, no. 5: 1751–1759. 10.1111/j.1365-2486.2010.02346.x.

[mec70451-bib-0024] Garcia‐Longoria, L. , S. Magallanes , X. Huang , et al. 2022. “Reciprocal Positive Effects on Parasitemia Between Coinfecting Haemosporidian Parasites in House Sparrows.” BMC Ecology and Evolution 22, no. 1: 73. 10.1186/s12862-022-02026-5. 35655150 PMC9164529

[mec70451-bib-0025] Geue, D. , and J. Partecke . 2008. “Reduced Parasite Infestation in Urban Eurasian Blackbirds ( *Turdus merula* ): A Factor Favoring Urbanization?” Canadian Journal of Zoology 86, no. 12: 1419–1425. 10.1139/Z08-129.

[mec70451-bib-0026] Glaizot, O. , L. Fumagalli , K. Iritano , F. Lalubin , J. V. Rooyen , and P. Christe . 2012. “High Prevalence and Lineage Diversity of Avian Malaria in Wild Populations of Great Tits ( *Parus major* ) and Mosquitoes ( *Culex pipiens* ).” PLoS One 7, no. 4: e34964. 10.1371/journal.pone.0034964. 22506060 PMC3323596

[mec70451-bib-0027] Gottdenker, N. L. , D. G. Streicker , C. L. Faust , and C. R. Carroll . 2014. “Anthropogenic Land Use Change and Infectious Diseases: A Review of the Evidence.” EcoHealth 11, no. 4: 4. 10.1007/s10393-014-0941-z. 24854248

[mec70451-bib-0028] Hellgren, O. , J. Waldenström , and S. Bensch . 2004. “A New PCR Assay for Simultaneous Studies of *Leucocytozoon*, *Plasmodium*, and *Haemoproteus* From Avian Blood.” Journal of Parasitology 90, no. 4: 797–802. 10.1645/GE-184R1. 15357072

[mec70451-bib-0029] Huijben, S. , D. G. Sim , A. N. William , and A. F. Read . 2011. “The Fitness of Drug‐Resistant Malaria Parasites in a Rodent Model: Multiplicity of Infection.” Journal of Evolutionary Biology 24, no. 11: 2410–2422. 10.1111/j.1420-9101.2011.02369.x. 21883612 PMC3304104

[mec70451-bib-0030] Karvonen, A. , J. Jokela , and A.‐L. Laine . 2019. “Importance of Sequence and Timing in Parasite Coinfections.” Trends in Parasitology 35, no. 2: 109–118. 10.1016/j.pt.2018.11.007. 30578150

[mec70451-bib-0031] Khasnis, A. A. , and M. D. Nettleman . 2005. “Global Warming and Infectious Disease.” Archives of Medical Research 36, no. 6: 689–696. 10.1016/j.arcmed.2005.03.041. 16216650

[mec70451-bib-0032] Kim, K. S. , and Y. Tsuda . 2012. “Avian Plasmodium Lineages Found in Spot Surveys of Mosquitoes From 2007 to 2010 at Sakata Wetland, Japan: Do Dominant Lineages Persist for Multiple Years?” Molecular Ecology 21, no. 21: 5374–5385. 10.1111/mec.12047. 23036191

[mec70451-bib-0033] Knowles, S. C. L. , M. J. Wood , R. Alves , T. A. Wilkin , S. Bensch , and B. C. Sheldon . 2011. “Molecular Epidemiology of Malaria Prevalence and Parasitaemia in a Wild Bird Population.” Molecular Ecology 20, no. 5: 1062–1076. 10.1111/j.1365-294X.2010.04909.x. 21073677

[mec70451-bib-0034] Lachish, S. , S. C. L. Knowles , R. Alves , M. J. Wood , and B. C. Sheldon . 2011a. “Fitness Effects of Endemic Malaria Infections in a Wild Bird Population: The Importance of Ecological Structure.” Journal of Animal Ecology 80, no. 6: 1196–1206. 10.1111/j.1365-2656.2011.01836.x. 21426343

[mec70451-bib-0035] Lachish, S. , S. C. L. Knowles , R. Alves , M. J. Wood , and B. C. Sheldon . 2011b. “Infection Dynamics of Endemic Malaria in a Wild Bird Population: Parasite Species‐Dependent Drivers of Spatial and Temporal Variation in Transmission Rates.” Journal of Animal Ecology 80, no. 6: 1207–1216. 10.1111/j.1365-2656.2011.01893.x. 21848864

[mec70451-bib-0036] Lalubin, F. , A. Delédevant , O. Glaizot , and P. Christe . 2013. “Temporal Changes in Mosquito Abundance ( *Culex pipiens* ), Avian Malaria Prevalence and Lineage Composition.” Parasites & Vectors 6, no. 1: 307. 10.1186/1756-3305-6-307. 24499594 PMC4029311

[mec70451-bib-0037] LaPointe, D. A. , C. T. Atkinson , and M. D. Samuel . 2012. “Ecology and Conservation Biology of Avian Malaria.” Annals of the New York Academy of Sciences 1249, no. 1: 211–226. 10.1111/j.1749-6632.2011.06431.x. 22320256

[mec70451-bib-0038] Liu‐Helmersson, J. , H. Stenlund , A. Wilder‐Smith , and J. Rocklöv . 2014. “Vectorial Capacity of *Aedes aegypti* : Effects of Temperature and Implications for Global Dengue Epidemic Potential.” PLoS One 9, no. 3: e89783. 10.1371/journal.pone.0089783. 24603439 PMC3946027

[mec70451-bib-0039] Loiseau, C. , R. J. Harrigan , C. Bichet , et al. 2013. “Predictions of Avian *Plasmodium* Expansion Under Climate Change.” Scientific Reports 3: 1. 10.1038/srep01126. PMC3553554 23350033

[mec70451-bib-0040] Loiseau, C. , T. Iezhova , G. Valkiūnas , et al. 2010. “Spatial Variation of Haemosporidian Parasite Infection in African Rainforest Bird Species.” Journal of Parasitology 96, no. 1: 1. 10.1645/GE-2123.1. 19860532

[mec70451-bib-0041] Otero, L. , J. J. Schall , V. Cruz , K. Aaltonen , and M. A. Acevedo . 2019. “The Drivers and Consequences of Unstable Plasmodium Dynamics: A Long‐Term Study of Three Malaria Parasite Species Infecting a Tropical Lizard.” Parasitology 146, no. 4: 453–461. 10.1017/S0031182018001750. 30319084

[mec70451-bib-0042] Paaijmans, K. P. , A. F. Read , and M. B. Thomas . 2009. “Understanding the Link Between Malaria Risk and Climate.” Proceedings of the National Academy of Sciences 106, no. 33: 13844–13849. 10.1073/pnas.0903423106. PMC2720408 19666598

[mec70451-bib-0043] Palinauskas, V. , G. Valkiūnas , C. V. Bolshakov , and S. Bensch . 2008. “ *Plasmodium relictum* (Lineage P‐SGS1): Effects on Experimentally Infected Passerine Birds.” Experimental Parasitology 120, no. 4: 372–380. 10.1016/j.exppara.2008.09.001. 18809402

[mec70451-bib-0044] Pérez‐Rodríguez, A. , S. Fernández‐González , I. de la Hera , and J. Pérez‐Tris . 2013. “Finding the Appropriate Variables to Model the Distribution of Vector‐Borne Parasites With Different Environmental Preferences: Climate Is Not Enough.” Global Change Biology 19, no. 11: 3245–3253. 10.1111/gcb.12226. 23606561

[mec70451-bib-0045] Perrin, A. , O. Glaizot , and P. Christe . 2022. “Worldwide Impacts of Landscape Anthropization on Mosquito Abundance and Diversity: A Meta‐Analysis.” Global Change Biology 28, no. 23: 6857–6871. 10.1111/gcb.16406. 36107000 PMC9828797

[mec70451-bib-0046] Perrin, A. , O. Glaizot , and P. Christe . 2025. “Migratory Birds Spread Their Haemosporidian Parasites Along the World's Major Migratory Flyways.” Oikos 2025: e11012.

[mec70451-bib-0047] Perrin, A. , A. Khimoun , A. Ollivier , et al. 2023. “Habitat Fragmentation Matters More Than Habitat Loss: The Case of Host–Parasite Interactions.” Molecular Ecology 32, no. 4: 951–969. 10.1111/mec.16807. 36461661

[mec70451-bib-0048] Perrin, A. , F. Schaffner , P. Christe , and O. Glaizot . 2023. “Relative Effects of Urbanisation, Deforestation, and Agricultural Development on Mosquito Communities.” Landscape Ecology 38, no. 6: 1527–1536. 10.1007/s10980-023-01634-w. 37229481 PMC10203030

[mec70451-bib-0049] Pigeault, R. , C.‐S. Cozzarolo , R. Choquet , et al. 2018. “Haemosporidian Infection and Co‐Infection Affect Host Survival and Reproduction in Wild Populations of Great Tits.” International Journal for Parasitology 48, no. 14: 1079–1087. 10.1016/j.ijpara.2018.06.007. 30391229

[mec70451-bib-0050] Pigeault, R. , C.‐S. Cozzarolo , J. Wassef , et al. 2024. “Spring Reproductive Success Influences Autumnal Malarial Load in a Passerine Bird.” Peer Community Journal 4: e18. 10.24072/pcjournal.378.

[mec70451-bib-0051] Råberg, L. , J. C. de Roode , A. S. Bell , P. Stamou , D. Gray , and A. F. Read . 2006. “The Role of Immune‐Mediated Apparent Competition in Genetically Diverse Malaria Infections.” American Naturalist 168, no. 1: 41–53. 10.1086/505160. 16874614

[mec70451-bib-0052] Richie, T. L. 1988. “Interactions Between Malaria Parasites Infecting the Same Vertebrate Host.” Parasitology 96, no. 3: 607–639. 10.1017/S0031182000080227. 3043327

[mec70451-bib-0053] Rivero, A. , and S. Gandon . 2018. “Evolutionary Ecology of Avian Malaria: Past to Present.” Trends in Parasitology 34, no. 8: 712–726. 10.1016/j.pt.2018.06.002. 29937414

[mec70451-bib-0054] Roiz, D. , S. Ruiz , R. Soriguer , and J. Figuerola . 2014. “Climatic Effects on Mosquito Abundance in Mediterranean Wetlands.” Parasites & Vectors 7, no. 333: 1–13. 10.1186/1756-3305-7-333. 25030527 PMC4223583

[mec70451-bib-0055] Rose, N. H. , M. Sylla , A. Badolo , et al. 2020. “Climate and Urbanization Drive Mosquito Preference for Humans.” Current Biology 30, no. 18: 3570–3579.e6. 10.1016/j.cub.2020.06.092. 32707056 PMC7511451

[mec70451-bib-0056] Samuel, M. D. , P. H. F. Hobbelen , F. DeCastro , et al. 2011. “The Dynamics, Transmission, and Population Impacts of Avian Malaria in Native Hawaiian Birds: A Modeling Approach.” Ecological Applications 21, no. 8: 2960–2973. 10.1890/10-1311.1.

[mec70451-bib-0057] Santiago‐Alarcon, D. , P. Carbó‐Ramírez , I. Macgregor‐Fors , C. A. Chávez‐Zichinelli , and P. J. Yeh . 2020. “The Prevalence of Avian Haemosporidian Parasites in an Invasive Bird Is Lower in Urban Than in Non‐Urban Environments.” Ibis 162, no. 1: 201–214. 10.1111/ibi.12699.

[mec70451-bib-0058] Santiago‐Alarcon, D. , I. MacGregor‐Fors , I. Falfán , et al. 2019. “Parasites in Space and Time: A Case Study of Haemosporidian Spatiotemporal Prevalence in Urban Birds.” International Journal for Parasitology 49, no. 3: 3. 10.1016/j.ijpara.2018.08.009. 30673588

[mec70451-bib-0059] Sehgal, R. N. M. , W. Buermann , R. J. Harrigan , et al. 2011. “Spatially Explicit Predictions of Blood Parasites in a Widely Distributed African Rainforest Bird.” Proceedings of the Royal Society B: Biological Sciences 278, no. 1708: 1025–1033. 10.1098/rspb.2010.1720. PMC3049032 20880888

[mec70451-bib-0060] Soares, L. , S. C. Latta , and R. E. Ricklefs . 2017. “Dynamics of Avian Haemosporidian Assemblages Through Millennial Time Scales Inferred From Insular Biotas of the West Indies.” Proceedings of the National Academy of Sciences 114, no. 25: 6635–6640. 10.1073/pnas.1702512114. PMC5488943 28607060

[mec70451-bib-0061] Suh, E. , M. K. Grossman , J. L. Waite , et al. 2020. “The Influence of Feeding Behaviour and Temperature on the Capacity of Mosquitoes to Transmit Malaria.” Nature Ecology & Evolution 4, no. 7: 7. 10.1038/s41559-020-1182-x. PMC7334094 32367033

[mec70451-bib-0062] Talla, C. , D. Diallo , I. Dia , et al. 2014. “Statistical Modeling of the Abundance of Vectors of West African Rift Valley Fever in Barkédji, Senegal.” PLoS One 9, no. 12: e114047. 10.1371/journal.pone.0114047. 25437856 PMC4250055

[mec70451-bib-0063] Tamayo‐Quintero, J. , M. San‐José , J. Martínez‐de la Puente , C. González‐Quevedo , and H. F. Rivera‐Gutierrez . 2025. “It's All About Scale: The Landscape Effect on Avian Haemosporidians.” Science of the Total Environment 962: 178426. 10.1016/j.scitotenv.2025.178426. 39813835

[mec70451-bib-0064] Valkiūnas, G. 2005. Avian Malaria Parasites and Other Haemosporidia. CRC press.

[mec70451-bib-0065] van Rooyen, J. , F. Lalubin , O. Glaizot , and P. Christe . 2013. “Avian Haemosporidian Persistence and Co‐Infection in Great Tits at the Individual Level.” Malaria Journal 12, no. 1: 40. 10.1186/1475-2875-12-40. 23360530 PMC3644249

[mec70451-bib-0066] White, A. 2015. Linking Human‐Disturbed Landscapes With Pathogen Prevalence in Wildlife: A Meta‐Analysis [PhD Thesis]. Colorado State University.

[mec70451-bib-0067] Wilkinson, L. C. , C. M. Handel , C. Van Hemert , C. Loiseau , and R. N. M. Sehgal . 2016. “Avian Malaria in a Boreal Resident Species: Long‐Term Temporal Variability, and Increased Prevalence in Birds With Avian Keratin Disorder.” International Journal for Parasitology 46, no. 4: 281–290. 10.1016/j.ijpara.2015.12.008. 26828894

[mec70451-bib-0068] Yan, W.‐L. , H.‐T. Sun , Y.‐C. Zhao , et al. 2024. “Global Prevalence of *Plasmodium* Infection in Wild Birds: A Systematic Review and Meta‐Analysis.” Research in Veterinary Science 168: 105136. 10.1016/j.rvsc.2024.105136. 38183894

