# Supplementary material for: Disentangling Environmental and Within‐Host Drivers of Parasite Dynamics in Natural Populations
Source: Mol Ecol. 2026 Jul 1;35(13):e70451. doi: 10.1111/mec.70451 (PMC13322711; doi:10.1111/mec.70451)
Supplement: Supplementary file 1 — Figure S1: Distribution of bird captures across sampling sites and years for the 245 recaptured birds infected by one of the three main lineages in our dataset (H. PARUS1 [H. majoris], P. SGS1 [ P. relictum ], and P. SW2 [P. homonucleophilum]). Figure S2: Distribution of bird captures across sampling sites and years for the 50 birds recaptured at least twice and infected by different haemosporidian lineages. [file MEC-35-e70451-s001.docx]

**Supplemental Information for:**

**Disentangling environmental and within-host drivers of parasite dynamics in natural populations**

Antoine Perrin, Heinz Richner, Molly Baur, Olivier Glaizot, and Philippe Christe

**Table of Contents:**

| **Figure S1** | Page 2 |
| --- | --- |
| **Figure S2** | Page 3 |

**Figure S1:** Distribution of bird captures across sampling sites and years for the 245 recaptured birds infected by one of the three main lineages in our dataset [H. PARUS1 (H. majoris), P. SGS1 (P. relictum), and P. SW2 (P. homonucleophilum)].

**Figure S2:** Distribution of bird captures across sampling sites and years for the 50 birds recaptured at least twice and infected by different haemosporidian lineages.
